# Assessment of Physical Activity by Wearable Technology During Rehabilitation After Cardiac Surgery: Explorative Prospective Monocentric Observational Cohort Study

**DOI:** 10.2196/mhealth.9865

**Published:** 2019-01-31

**Authors:** Isabeau Thijs, Libera Fresiello, Wouter Oosterlinck, Peter Sinnaeve, Filip Rega

**Affiliations:** 1 Research Unit of Cardiac Surgery Department of Cardiovascular Sciences University Hospitals Leuven Leuven Belgium; 2 Department of Cardiac Surgery Katholiek Universiteit Leuven Leuven Belgium; 3 Institute of Clinical Physiology National Research Council Pisa Italy; 4 Research Unit of Cardiology Department of Cardiovascular Sciences University Hospitals Leuven Leuven Belgium

**Keywords:** fitness trackers, coronary artery bypass, cardiac surgery, cardiac rehabilitation, postoperative care, wearable, physical activity, exercise

## Abstract

**Background:**

Wearable technology is finding its way into clinical practice. Physical activity describes patients’ functional status after cardiac surgery and can be monitored remotely by using dedicated trackers.

**Objective:**

The aim of this study was to compare the progress of physical activity in cardiac rehabilitation by using wearable fitness trackers in patients undergoing coronary artery bypass surgery by either the conventional off-pump coronary artery bypass (OPCAB) or the robotically assisted minimally invasive coronary artery bypass (RA-MIDCAB). We hypothesized faster recovery of physical activity after RA-MIDCAB in the first weeks after discharge as compared to OPCAB.

**Methods:**

Patients undergoing RA-MIDCAB or OPCAB were included in the study. Each patient received a Fitbit Charge HR (Fitbit Inc, San Francisco, CA) physical activity tracker following discharge. Rehabilitation progress was assessed by measuring the number of steps and physical activity level daily. The physical activity level was calculated as energy expenditure divided by the basic metabolic rate.

**Results:**

A total of 10 RA-MIDCAB patients with a median age of 68 (min, 55; max, 83) years and 12 OPCAB patients with a median age of 69 (min, 50; max, 82) years were included. Baseline characteristics were comparable except for body mass index (RA-MIDCAB: 26 kg/m²; min, 22; max, 28 versus OPCAB: 29 kg/m²; min, 27; max, 33; *P*<.001). Intubation time (*P*<.05) was significantly lower in the RA-MIDCAB group. A clear trend, although not statistically significant, was observed towards a higher number of steps in RA-MIDCAB patients in the first week following discharge.

**Conclusions:**

RA-MIDCAB patients have an advantage in recovery in the first weeks of revalidation, which is reflected by the number of steps and physical activity level measured by the Fitbit Charge HR, as compared to OPCAB patients. However, unsupervised assessment of daily physical activity varied widely and could have consequences with regard to the use of these trackers as research tools.

## Introduction

Kolesov V (1964) performed the first coronary artery bypass graft (CABG) using the internal mammary artery to treat a patient with ischemic myocardial heart disease [1]. Off-pump coronary artery bypass (OPCAB) surgery was developed to reduce potential adverse effects induced by the use of cardiopulmonary bypass and cardioplegic arrest [2]. In this approach, the harvesting of the internal mammary arteries and anastomoses are performed on a beating heart through a median sternotomy.

Robotically assisted minimally invasive direct coronary artery bypass (RA-MIDCAB) aims to further reduce the invasiveness of the OPCAB approach by avoiding midline sternotomy. In RA-MIDCAB surgery, the internal mammary arteries are prelevated via a thoracoscopy using robotic assistance. The grafting of the bypass is performed in a second stage via a small (4-5 cm) left anterolateral minithoracotomy. Similar to OPCAB, the anastomosis is performed on a beating heart, without the use of cardiopulmonary bypass. The RA-MIDCAB approach reduces morbidity, length of hospital stay, need for blood transfusion, and wound infections [3-5]. The time for recovery is controversial among studies [3-5]: Some indicate an earlier recovery to full physical activity [3,6].

After cardiac surgery, patients need structured support to improve functional capacity and restore their quality of life. Phase II cardiac rehabilitation programs are developed to deliver comprehensive support such as monitoring in physical and psychological conditions and education of patients on healthy long-term routines. Phase II cardiac rehabilitation is suggested as a class I recommendation in the treatment of cardiac diseases by the European Society of Cardiology, the American Heart Association, and the American College of Cardiology [7-9]. Adherence could potentially be tracked by the use of remote monitoring systems.

Physical activity or fitness trackers are wearable sensors, often worn as a wristband or embedded in a smartwatch or mobile phone, that collect data on one’s daily physical activity. Most of these commercially available trackers include an accelerometer to assess step counts; distance walked; and intensity, duration, and type of movement (eg, walking, running, and jogging). Thus, users can have direct access to their personal data and take an active role in monitoring their health [10,11].

These trackers are also of use in clinical practices and research. Accelerometry data can be derived noninvasively and in unsupervised, free-living conditions, which provides an opportunity to better describe patients’ activity of daily living and health status in terms of mobility, behavioral pattern, and functional ability. Consequently, these data can contribute to more comprehensive, relevant, and high-quality clinical research data [10]. In clinical practices, home telemonitoring trials show favorable results in pulmonary and cardiac patients [12]. In cardiac rehabilitation, multiple cardiac telecare trials have shown a noninferiority or superiority of telemonitoring and telecoaching of patients in a cardiac rehabilitation program compared to conventional center-based supervised cardiac rehabilitation programs [12,13,14]. These physical activity trackers have the ability to encourage exercise and lifestyle behavior and monitor and share progress [11,12,15]. As such, wearables could potentially have a future in at-home management and remote monitoring of patients with chronic diseases and in secondary preventive care after cardiac surgery.

At the University Hospital of Leuven, an explorative clinical observational study was performed to evaluate physical activity in patients after coronary artery bypass (CAB) surgery. This study aimed to quantify physical fitness at particular time points and investigate whether minimal-access surgical procedures can assure faster recovery and better outcome than the conventional, more invasive surgical procedure.

## Methods

### Study Design

#### Protocol

An explorative prospective monocentric observational cohort study was conducted at the Cardiac Surgery Department of the University Hospitals of Leuven. The clinical protocol conformed to the principles outlined in the Declaration of Helsinki and was approved by the ethical committee of the University Hospitals Leuven. All patients provided written informed consent prior to inclusion in the study.

#### Patients

The study included patients with coronary artery disease who were eligible for elective surgical revascularization according to the most recent guidelines of the European Society of Cardiology [16]. They were scheduled to undergo either an RA-MIDCAB or OPCAB procedure. In both approaches, grafts are anastomosed on the diseased vessels without the use of cardiopulmonary bypass—the so-called off-pump technique. In OPCAB, a full sternotomy is performed, whereas in RA-MIDCAB, the anastomosis is performed through a small left anterolateral thoracotomy. Additionally, in RA-MIDCAB, internal mammary arteries (used as grafts) are harvested using robotic assistance from the Da Vinci Surgical System (Intuitive Surgical Inc, Sunnyvale, CA).

#### Eligibility Criteria

The main exclusion criteria were urgently scheduled and on-pump procedures, mobility problems that could interfere with physical activity, and the presence of cognitive impairment that prevented subjects from fully understanding the protocol. An overview of the inclusion and exclusion criteria is provided in Multimedia Appendix 1.

**Figure 1:**
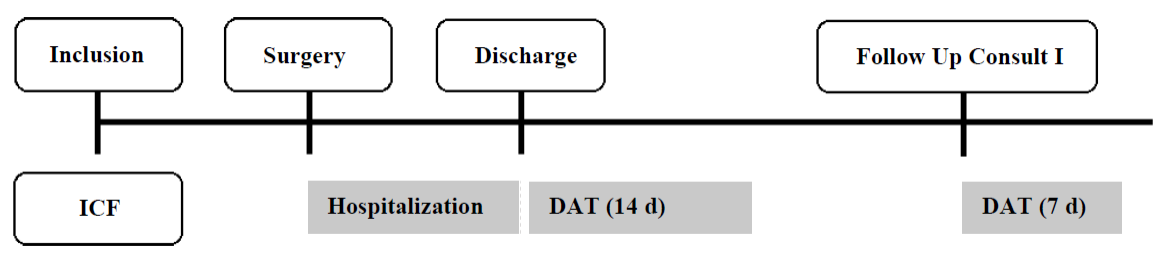
Timeline of the study design constructed in three evaluation time points: a preoperative inclusion, a 14-day Fitbit-wearing period after discharge, and a 7-day Fitbit-wearing period after a follow-up consult, 4 weeks after discharge. ICF: Informed Consent Form; DAT: Daily Activity Tracking.

#### Evaluation Design

The protocol was organized in three evaluation time points: a preoperative baseline assessment and two periods of wearing a physical activity tracker (Figure 1). At discharge, patients received a Fitbit Charge HR (Fitbit Inc, San Francisco, CA) and were asked to wear the wearable device for 14 consecutive days. Four weeks later, a follow-up consultation was scheduled, and the patient was again asked to wear the device for 7 days. The patients were asked to return the tracker to the hospital by mail. Additionally, throughout the hospital stay, clinical data were collected. An overview of the variables for which data were collected is shown in Multimedia Appendix 2.

### Physical Fitness Assessment

Subjects’ daily physical activity was described by parameters recorded by the Fitbit Charge HR. This activity tracker is a wrist band with an interface through which patients can monitor their real-time progress. The tracker was set according to the height, weight, and age of the subject. Subjects were instructed to wear the wristband as much as possible during the day. The tracker records the daily number of steps, the distance walked, the flights of stairs taken, the intensity and duration of exercise, the estimated energy expenditure, the sleeping pattern and the heart rate variation using the Pure Pulse technology (FitBit Inc).

### Data Analysis

Data from the Fitbit Charge HR were analyzed by calculating the weekly average step counts and energy expenditure (kcal). For energy expenditure, the physical activity level was calculated by dividing the total caloric expenditure by the basic metabolic rate. This physical activity level represents the physical activity adjusted for weight, height, and age (included in the basic metabolic rate). For every subject, the first (discharge) and last (return) day of Fitbit wearing were excluded, since biased results were expected. Furthermore, continuous heart rate was evaluated to check periods when patients did not wear the device. Up to 2 hours a day of non-wearing time were neglected; if the non-wearing period was longer, that day was excluded. This time loss could be due to battery charging or activities such as bathing or showering.

### Outcome Measures

#### Primary Outcome

The primary outcome was the objective physical activity score described by the Fitbit activity tracker data during the two periods mentioned above in Evaluation Design. Weekly average number of steps and weekly average physical activity level were used to quantify physical activity.

#### Secondary Outcomes

Secondary outcomes included observational data including demography, cardiac and noncardiac history, operative variables, and postoperative complications until 4 weeks after discharge.

### Statistical Analyses

Statistical analyses were carried out using IBM SPSS Statistics, Version 24 (IBM Corp, Armonk, NY). Since only a small sample size was included, differences in continuous variables were analyzed using two-sided Mann Whitney *U* tests and reported as median and range with minimal and maximal values. For dichotomous variables, a Fisher exact test was performed, and for categorical variables, a Mann Whitney *U* test was performed. For repeated measures analysis, the nonparametric Friedman *t* test was used. Statistical significance was considered for *P* values<.05.

## Results

### Patient Recruitment

**Figure 2:**
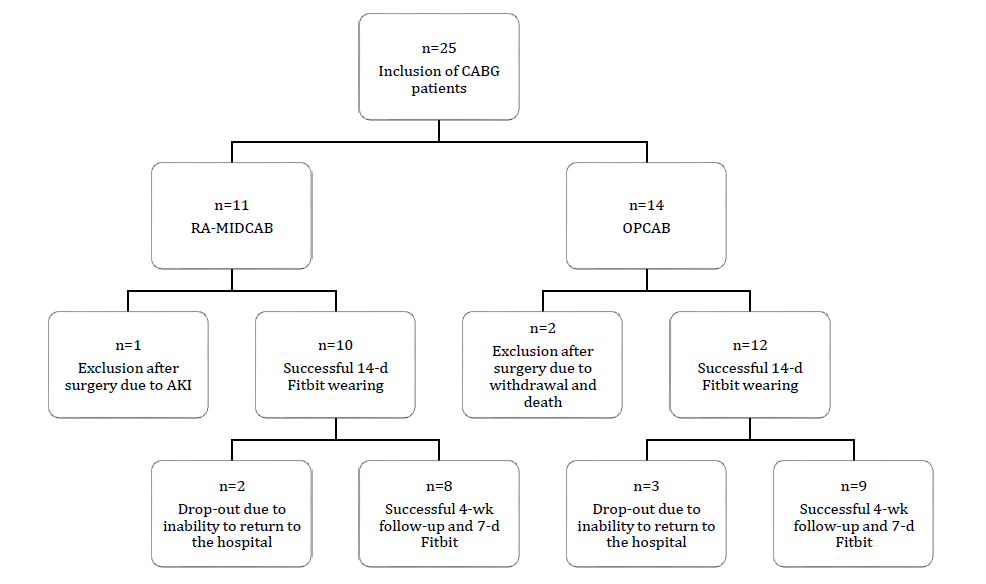
Study flowchart. CABG: coronary artery bypass graft; RA-MIDCAB: robotically assisted minimally invasive direct coronary artery bypass; OPCAB: off-pump coronary artery bypass; AKI: acute kidney injury.

Patients were recruited from January 2017 to April 2017. In total, 25 patients were enrolled, of which 11 were RA-MIDCAB patients and 14 were OPCAB patients. Three patients were excluded after the surgery: one patient was excluded from the RA-MIDCAB group due to a prolonged hospital stay as a consequence of acute on chronic kidney failure, and two patients dropped out after surgery in the OPCAB group (one withdrew from the study and one died). Furthermore, after the 14-day Fitbit-wearing period, three patients in the OPCAB group and two patients in the RA-MIDCAB group dropped out. A study flowchart is presented in Figure 2. Results of the baseline characteristics are listed in Table 1.

The two groups did not significantly differ in age (*P*=.79), gender (*P*=>.99), height (*P*=.79), and weight (*P*=.07), but OPCAB patients had a significantly higher body mass index (29 kg/m²) than RA-MIDCAB patients (26 kg/m²; *P*<.001).

Heart failure distribution according to the New York Heart Association class was not significantly different in both groups (*P*=.89), and most subjects belonged to class I and II. The median left ventricular ejection fraction was 60% (min, 45; max, 78) in the RA-MIDCAB group and 60% (min, 40; max, 78) in the OPCAB group. Furthermore, both groups showed a similar distribution in the European System for Cardiac Operative Risk Evaluation (EuroSCORE II; *P*=.21), left ventricular ejection fraction (*P*=.69), history of arrhythmias (*P*=.46), history of myocardial infarction (*P*=.90), and mitral regurgitation (*P*>.99).

There was a trend towards the presence of hypercholesterolemia in OPCAB patients compared to RA-MIDCAB patients (92% and 50%, respectively, *P*=.06). No significant differences were observed in the presence of comorbidities (Table 1).

Intubation time was significantly higher in the OPCAB group (*P*<.05), with 15 hours 30 minutes (min, 8 hours 2 minutes; max, 21 hours 50 minutes) in contrast to 8 hours 45 minutes (min, 5 hours 49 minutes; max, 23 hours) in the RA-MIDCAB group (Table 1). In addition, the number of grafts was significantly higher in the OPCAB group (*P*<.005), and the operation duration was higher in the OPCAB group (*P*=.10). The median duration was 5 hours 15 minutes (min, 3 hours 7 minutes; max, 6 hours 58 minutes), whereas the median duration in the RA-MIDCAB group was 4 hours 40 minutes (min, 3 hours 18 minutes; max, 5 hours 26 min).

Postoperatively, five patients in the RA-MIDCAB group and one patient in the OPCAB group were transferred to the postanesthetic care unit; a trend towards significance was observed in this parameter (*P*=.06). The length of stay at the postoperative care units (including the postanesthetic care unit and intensive care unit) was significantly lower in the RA-MIDCAB group (*P*<.001), with a median stay of 20 hours 45 minutes (min, 15 hours 30 minutes; max, 45 hours) in this group and 30 hours 45 minutes (min, 18 hours 30 minutes; max, 77 hours) in the OPCAB group. Furthermore, the overall hospital stay was similar in both groups (*P*=.21).

**Table 1:** Baseline characteristics and postoperative parameters.

Characteristics			RA-MIDCAB^a^ (n=10)	OPCAB^b^ (n=12)		*P* value	
**Demographics**							
	Sex (male), n (%)		9 (90)	10 (78)		>.99	
	Age (y), median (min, max)		68 (55, 83)	69 (50, 82)		.79	
	Height (cm), median (min, max)		172 (154, 178)	171 (155, 178)		.79	
	Weight (kg), median (min, max)		77 (57, 90)	83 (75, 100)		.07	
	Body mass index (kg/m^²^), median (min, max)		26 (22, 28)	29 (27, 33)		<.005^c^	
**Cardiac history**							
	**NYHA^d^ Class, n**					.89	
		I	4		5		
		II	4		5		
		III	2		2		
	Presence of unstable angina, n		2	3		.79	
	Left ventricular ejection fraction (%), median (min, max)		60 (45, 78)	60 (40, 78)		.69	
	EuroSCORE II^e^ (%), median (min, max)		1.7 (0.6, 5.5)	1.1 (0.6, 2.8)		.21	
	History of arrhythmias, n		1	0		.46	
	**History of myocardial infarction, n**					.9	
		Non-ST-segment elevated myocardial infarction, n	2		3		
		ST-segment elevated myocardial infarction, n	1		0		
	Mild mitral regurgitation, n		5	5		>.99	
**Non-cardiac history**							
	**Smoking status, n**					.64	
		Ex-smoker since >1 month	5		6		
		Smoker	3		1		
	Diabetes mellitus type II, n		3	2		.62	
	Arterial hypertension, n		7	10		.62	
	Pulmonary hypertension, n^f^		1	1		>.99	
	Hypercholesterolemia, n		5	11		.06	
	Renal impairment, n		9	6		.83	
	Peripheral vascular diseases, n		1	2		>.99	
	Chronic obstructive pulmonary disease, n		1	2		>.99	
**Operative data,** median (min, max)							
	Operation duration, min		281 (198, 326)	316 (187, 418)		.1	
	Intubation time, min		527 (349, 1380)	931 (482, 1310)		<.05^c^	
	Number of grafts, n		2 (1, 2)	3.5 (2, 4)		<.005^c^	
**Postoperative data**							
	Postanesthetic care unit stay, n		5	1			
	Postoperative care unit length of stay (min), median (min, max)		1245 (930, 2700)	1440 (1110, 4620)		<.001^c^	
	Hospital length of stay (d), median (min, max)		6 (4, 12)	7 (5, 15)		.21	
**Complications, n**							
	Wound infections		0	2		.48	
	Pulmonary infections		0	2		.48	
	Pleural effusions		2	3		>.99	
	New arrhythmias		2	1		.57	
	Hypokalemia		4	3		.65	
	Pericarditis		2	0		.2	

^a^RA-MIDCAB: robotically assisted minimally invasive direct coronary artery bypass.

^b^OPCAB: off-pump coronary artery bypass.

^c^*P* values are significant.

^d^NYHA: New York Heart Association functional classification of heart failure.

^e^EuroSCORE II: European System for Cardiac Operative Risk Evaluation.

^f^Pulmonary hypertension was defined as pulmonary pressure>25 mmHg.

**Table 2:** Step count analysis recorded by Fitbit Charge HR in RA-MIDCAB and OPCAB patients.

Average step counts		RA-MIDCAB^a^	Percentage^b^	OPCAB^c^	*P* value
**Overall analysis, median (min, max), n**					
	Week 1	3715 (1637, 6720), 10	335	1110 (739, 10,195), 11	.06
	Week 2	4357 (1415, 7671), 10	238	1832 (856, 11,282), 10	.33
	Week 5	6012 (3473, 11579), 8	105	5719 (2128, 11,948), 9	.7
**Analysis without dropouts, median (min, max), n**					
	Week 1	3715 (1734, 6720), 8	371	1001 (739, 10,195), 9	.07
	Week 2	4357 (1512, 7286), 8	459	949 (856, 11,282), 9	.17
	Week 5	6012 (3473, 11579), 8	105	5719 (2128, 11,948), 9	.7
**Repeated measures** **Friedman** ***t*** **test**					
Chi-square		28		30	—^d^
*P* value		<.001		<.001	—

^a^RA-MIDCAB: robotically assisted minimally invasive direct coronary artery bypass.

^b^Percentage of number of steps in RA-MIDCAB patients compared to OPCAB patients.

^c^OPCAB: off-pump coronary artery bypass.

^d^Not applicable.

### Primary Outcome

#### Step counts

Data were corrected for periods when the physical activity tracker was not worn. In week 1, a total of 3 days for one patient were excluded from the analysis. In week 2, a total of 9 days, distributed over four patients, were excluded. In week 5, 2 days, distributed over two patients, were excluded.

**Figure 3:**
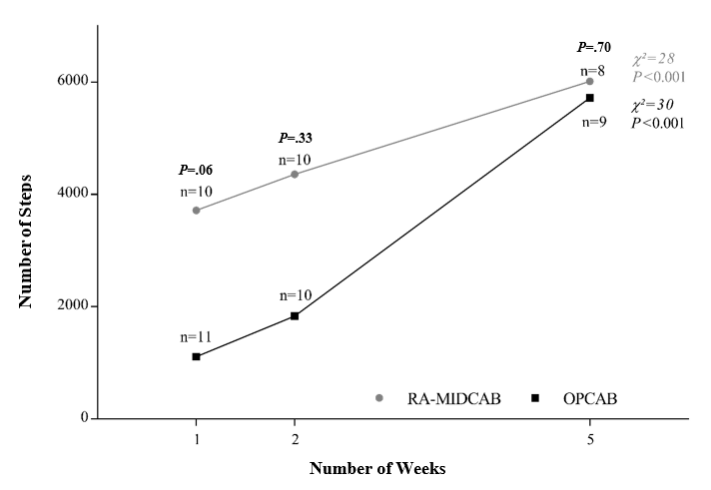
Weekly average number of steps in robotically assisted minimally invasive direct coronary artery bypass (RA-MIDCAB) and off-pump coronary artery bypass (OPCAB) patients plotted over time. Weekly average step count is plotted as median over time. n indicates the number of patients included in the cohort result. *P* value is for the Mann Whitney *U* test for the difference between the two groups at that time point. *Χ^2^* results of repeated measures Friedman *t* test.

**Figure 4:**
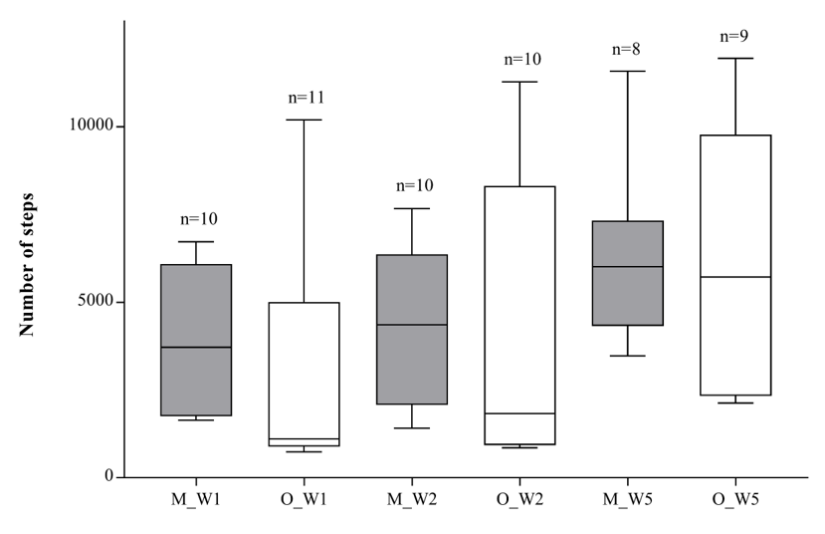
Boxplots of weekly average number of steps in robotically assisted minimally invasive direct coronary artery bypass (RA-MIDCAB) and off-pump coronary artery bypass (OPCAB) patients. Weekly average step counts is shown as box and whisker plots, presenting medians, 25% and 75% quartiles, minimums, and maximums. n indicates the number of patients included in the cohort result. M_W1: MIDCAB result in week 1; O_W1: OPCAB results in week 1; M_W2: MIDCAB results in week 2; O_W2: OPCAB results in week 2; M_W5: MIDCAB results in week 5; O_W5: OPCAB results in week 5.

In the first week, the RA-MIDCAB group showed a higher average number of steps than the OPCAB group, a result almost statistically significant (*P*=.06). Similarly, in the second week after discharge, RA-MIDCAB patients took more steps, but no significant difference was observed between the groups (*P*=.33). In week 5, the OPCAB group bridged the gap in the number of steps, and the average number of steps was similar between the two groups (*P*=.70; Table 2; Figures 3 and 4).

A nonparametric Friedman *t* test was performed to analyze the repeated measures analysis for the number of steps over time. A significant change over time was observed in the RA-MIDCAB group (28 steps; *P*<.001) and the OPCAB group (30 steps; *P*<.001; Table 2).

#### Physical Activity Level

With regard to the physical activity level, no significant differences were observed in weeks 1, 2, and 5 between the RA-MIDCAB and OPCAB groups (*P*=.36, *P*=.36, and *P*=.50, respectively). However, the physical activity level was higher in the RA-MIDCAB group than in the OPCAB group in all weeks (Table 3).

**Table 3:** Physical activity level^a^ analysis recorded by Fitbit Charge HR in RA-MIDCAB and OPCAB patients.

Time point		RA-MIDCAB^b^		OPCAB^c^		*P* value
		median (min, max)	n	median (min, max)	n	
**Overall analysis**						
	Week 1	1.39 (1.05, 1.71)	10	1.29 (1.08, 1.59)	11	.36
	Week 2	1.41 (1.04, 1.63)	10	1.32 (1.04, 1.60)	10	.36
	Week 5	1.52 (1.13, 1.90)	8	1.44 (1.16, 1.80)	9	.5
**Analysis without dropouts**						
	Week 1	1.39 (1.05, 1.71)	8	1.26 (1.08, 1.59)	9	.4
	Week 2	1.41 (1.04, 1.63)	8	1.23 (1.04, 1.60)	9	.42
	Week 5	1.52 (1.13, 1.90)	8	1.44 (1.16, 1.80)	9	.5

^a^Physical activity level calculated as total energy expenditure divided by basic metabolic rate.

^b^RA-MIDCAB: robotically assisted minimally invasive direct coronary artery bypass.

^c^OPCAB: off-pump coronary artery bypass; data are reported as median (min, max).

## Discussion

### Principal Findings

We evaluated physical activity in cardiac rehabilitation by using the Fitbit Charge HR tracker device after conventional and minimally invasive CAB surgery. A clear trend was observed towards a higher physical activity level in RA-MIDCAB patients than in OPCAB patients, which was reflected in the number of steps and physical activity level, although statistical significance was not reached.

### Value of Wearable Activity Trackers in Surgical Outcome Research

To our knowledge, this is the first study to use wearable activity tracking in a clinical environment to compare the outcome of two types of cardiac surgery interventions. The Fitbit Charge HR provided useful information about patients’ physical activity in this study. Wearable activity trackers are finding their way in research and medical practices [10,15,17]. An important limitation, however, is that commercially available activity trackers are often not thoroughly validated for their accuracy and reliability. Studies showed that step count accuracy is dependent on gait patterns in healthy volunteers [18,19]. In the elderly and chronically ill, a negative correlation was found between the gait pattern and step count accuracy, as assessed by other commercially available activity trackers [18,20]. Postsurgical patients who are still in recovery and probably walk at a slow speed will create a bias in the number of steps counted. In addition, the Fitbit Charge HR is still rarely used in research. However, older-generation models have been tested for their step count accuracy [21-24] and energy expenditure estimation [25,26].

Taking into account the limitations of the Fitbit technology, it is worth highlighting that our data were not analyzed as exact results but were only used to compare the RA-MIDCAB and OPCAB groups. Any error in the step count technology would affect RA-MIDCAB and OPCAB patients in a similar way without impacting the comparative analysis performed in this work. No baseline references are available, and Fitbit does not disclose algorithms or mean error values. Hence, it was not possible to apply mean error corrections. However, it is distinctively true that further research is needed before commercially available self-monitoring wearables can be used in clinical applications.

Besides the lack of validation, wearable activity trackers have a promising future. Activity trackers provide the possibility for patients to monitor their activity patterns and share their progress with physicians, friends, and family members. Therefore, these trackers can be used as motivational tools to reach and maintain a healthy active lifestyle [15]. In this study, subjects were nearly always compliant and motivated to wear the tracker and monitor their own progress. Physical activity is one of the most health-enhancing practices, especially in primary and secondary prevention of cardiovascular risk factors. Physical activity counselling (by use of wearable activity monitors) has been shown to improve healthy lifestyles [27-29]. Savage et al (2008) found significant correlations between the daily number of steps in the first weeks of Phase II cardiac rehabilitation and cardiovascular risk factors [30].

The future for this wearable activity trackers is still unknown, but its implementation in medical practice would provide many benefits, for instance, in cardiac rehabilitation to overcome barriers to cardiac rehabilitation programs. Multiple trials have shown that wearable activity tracking and digital health devices encourage patients to improve their physical behavior and are therefore useful tools in cardiac rehabilitation. The Telerehab III trial showed that telerehabilitation, through use of a commercially available accelerometer, provided substantial and persistent health benefits and novel, cost-efficient care [31].

### Comparison of Minimally Invasive and Conventional Coronary Artery Bypass

Our study showed that both groups were comparable. No significant differences were found in baseline characteristics, except for body mass index and the number of anastomosed grafts. EuroSCORE II calculation showed no significant difference in the predicted operative mortality. OPCAB patients have a more pronounced disease and may be considered more unwell, which can be reflected in the body mass index. In this group of patients, however, it seems that body mass index does not have a significant influence on physical activity and performance. Spearman correlation analysis showed that weight/body mass index was not related with the number of steps at any time point (Multimedia Appendix 3). Surgical and postsurgical data reflect the difference between both procedures. RA-MIDCAB entails a shorter intubation time and a shorter stay at postoperative care units, with more patients transferred to the postanesthetic care unit postoperatively (fast-track treatment). These findings are in line with previous studies [4,32]. Hospital length of stay, however, showed no significant difference between the two study groups; this is in contrast to the findings of other studies [33-35], which could be due to the small sample size of the present study. However, this variable might be dependent on institutional protocols and decision making of physicians and surgeons [36].

Despite the similar length of stay and baseline characteristics between the two groups, a clear trend was observed toward higher physical activity reflected in step counts and physical activity level in RA-MIDCAB patients in the first weeks, although statistical significance was not reached. Step counts depict the actual daily walking of patients during the day. The physical activity level depicts the energy expenditure as a result of activity, adjusted for individual basic metabolic rate. Therefore, both parameters interpret physical activity in a different manner and should be interpreted accordingly. It is harder to reach significance in the physical activity level analysis due to the smaller scale of variations. Owing to its explorative nature, this study is probably underpowered to detect smaller differences and is influenced greatly by outliers. Both groups showed significant changes in the number of steps over time (Table 2). Together with the differences between the groups, this could indicate that RA-MIDCAB patients advance in the early stages and OPCAB patients need some time to catch up. It must be noted that physical activity varied greatly among subjects, which could be due to the accuracy levels and algorithms of the device itself. In addition, physical activity is dependent on personal habits and character, referred to as self-efficacy [37,38], and the motivational support from the environment (relatives and friends). Patients who are sedentary before the surgery would likely abide by this lifestyle after surgery. Patients who are regularly active would probably be more motivated to achieve their prior level of fitness before the disease became symptomatic. This was illustrated in the Telerehab III trial where patients partially relapsed after telerehabilitation was stopped [31]. However, this effect would influence both groups similarly. Furthermore, inclusion in a clinical study and the intervention for monitoring activity by use of a tracker could be motivating factors. The tracker makes it possible for patients to monitor their own progress and activity. These factors could possibly also contribute to achieving a higher level of activity.

Both groups of patients walked about 5000 to 6000 steps a day at steady state in the fifth week after discharge. The American Heart Association recommends that healthy subjects walk 10,000 steps a day for overall better health outcomes, including cardiovascular outcome [39]. The official recommendation by the American Heart Association and World Health Organization is 150 minutes of moderate-intensity aerobic physical activity a day for 5 days a week [40], equivalent to 7000-8000 steps a day. Prior studies evaluating the number of steps in patients with coronary artery disease in secondary prevention proposed a target of 7500 steps a day to correlate with improved condition in terms of lipid profiles, muscle endurance, and body mass index [41,42].

### Limitations

This explorative observational study has multiple limitations. The lack of validation for this wearable technology was already described in the Discussion section.

Subjects were scheduled for either OPCAB or RA-MIDCAB surgery based on the coronary anatomy (number of grafts), comorbidities, and endovascular options. Therefore, they were matched according to baseline characteristics, cardiac history, and comorbidity profile, but the groups were found to be significantly different for body mass index, which was higher in the OPCAB group. Although body mass index was not significantly correlated to the number of steps at any point of the study, it might still be an influencing factor for physical activity. However, the difference was not taken into account in further analysis.

As stated above, physical activity is influenced by other factors in addition to the impact of a surgical intervention. Not all patients are equally active in nature and the differences may depend on self-efficacy, the choice for physiotherapy, and cardiac rehabilitation thereafter. Physiotherapy in the first weeks after surgery and the following Phase II cardiac rehabilitation may significantly influence the progress in physical activity.

Subjects were recommended to wear the activity tracker all day and to take it off only for charging or while showering. To verify if patients were constantly wearing the device, we checked for any missing data in continuous heart rate monitoring. Apart from this measure, it was difficult to supervise the wearing time. In an ideal setting, these patient cohorts would be analyzed in a randomized controlled trial. In addition, the present study is based on a small sample size. Hence, the results should be interpreted with caution, and further investigations should be carried out before outlining definitive conclusions.

### Conclusions

This research aimed to evaluate postsurgical cardiac rehabilitation progress by using commercially available wearable technology. We confirm our hypothesis that RA-MIDCAB patients have an advantage over OPCAB patients with regard to revalidation. Although not statistically significant, the RA-MIDCAB patient cohort showed a clear trend towards higher physical activity level in the first weeks after surgery. The exact hinge point must be confirmed with a larger number of patients. This work highlighted the feasibility of the use of wearable technology for physical activity monitoring in a clinical setting. Further research should be conducted to evaluate the accuracy and reliability of wearable technology before it serves clinical applications, especially in nonhealthy subjects with an altered gait pattern.

## Appendix

Multimedia Appendix 1 Inclusion and exclusion criteria.

Multimedia Appendix 2 Baseline characteristics and demographics.

Multimedia Appendix 3 Spearman correlations with the number of steps in weeks 1, 2, and 5.

## Abbreviations

CAB: coronary artery bypass
CABG: coronary artery bypass grafting
EuroSCORE II: European System for Cardiac Operative Risk Evaluation
NYHA: New York Heart Association functional classification of heart failure
OPCAB: off-pump coronary artery bypass
RA-MIDCAB: robotically assisted minimally invasive direct coronary artery bypass
STEMI: ST-segment elevated myocardial infarction
