# Supplementary material for: Assessment of Physical Activity by Wearable Technology During Rehabilitation After Cardiac Surgery: Explorative Prospective Monocentric Observational Cohort Study
Source: JMIR Mhealth Uhealth. 2019 Jan 31;7(1):e9865. doi: 10.2196/mhealth.9865 (PMC6374731; doi:10.2196/mhealth.9865)
Supplement: Multimedia Appendix 1 [file mhealth_v7i1e9865_app1.pdf]

Appendix 1: Table I: Inclusion and exclusion criteria

Inclusion criteria	• Informed written consent<br>• ≥ 18 years old<br>• Undergoing a coronary artery bypass graft surgery via:<br>o A median sternotomy approach, meaning full sternotomy, prelevation of the left and/or right internal mammary arteries, followed by graft anastomosis without use of cardiopulmonary bypass (so-called off pump).<br>o Robotic harvesting of the left internal mammary artery, followed by a small left antero-lateral thoracotomy, via which the internal mammary artery is anastomosed, also without use of cardiopulmonary bypass (so-called off pump)<br>• All anti-coagulant medication is stopped except for Asaflow®
Exclusion criteria	• On-pump coronary artery bypass surgery<br>• Urgent procedures<br>• Concomitant valvular disease<br>• Previous cardiac surgery<br>• Mobility problems that could prevent being physically active<br>• Mental or cognitive impairments
