# Supplementary material for: Assessment of Physical Activity by Wearable Technology During Rehabilitation After Cardiac Surgery: Explorative Prospective Monocentric Observational Cohort Study
Source: JMIR Mhealth Uhealth. 2019 Jan 31;7(1):e9865. doi: 10.2196/mhealth.9865 (PMC6374731; doi:10.2196/mhealth.9865)
Supplement: Multimedia Appendix 2 [file mhealth_v7i1e9865_app2.pdf]

Appendix 2: Table II: Baseline characteristics and demographics.

Demographics	Sex<br>Date of birth<br>Age<br>Body Mass Index<br>Weight<br>Length
Cardiac history	NYHA class[a]<br>Stable or Unstable Angina<br>Left Ventricular Ejection fraction<br>EuroSCORE II[b]<br>History of arrhythmias<br>• Ventricular<br>• Atrial<br>History of myocardial infarction<br>Congestive cardiac failure<br>Mitral regurgitation
Non-cardiac history	Smoking status<br>• Non-smoker<br>• Ex-smoker since > 1 month<br>• Smoker<br>Diabetes Mellitus<br>Arterial Hypertension<br>Pulmonary Hypertension<br>Hypercholesterolemia/ Hyperlipidaemia<br>Hyperthyroidism<br>Cerebrovascular disease:<br>• Cerebrovascular attack<br>• Transient ischaemia attack<br>Peripheral vascular disease<br>Chronic Obstructive Pulmonary Disease<br>Renal impairment

[a]NYHA Class: New York Heart Association Functional Classification for Heart Failure; [b]EuroSCORE II: The European System for Cardiac Operative Risk Evaluation
