# Supplementary material for: Assessment of Physical Activity by Wearable Technology During Rehabilitation After Cardiac Surgery: Explorative Prospective Monocentric Observational Cohort Study
Source: JMIR Mhealth Uhealth. 2019 Jan 31;7(1):e9865. doi: 10.2196/mhealth.9865 (PMC6374731; doi:10.2196/mhealth.9865)
Supplement: Multimedia Appendix 3 [file mhealth_v7i1e9865_app3.pdf]

Appendix 3: Table III: Spearman's correlations with number of steps in week 1, 2 and 5.

	**Week 1 average steps**		**Week 2 average steps**		**Week 5 average steps**	
	Spearman's Rho	*P* value	Spearman's Rho	*P* value	Spearman's Rho	*P* value
Baseline characteristics						
Body Mass Index	-0.24	0.30	-0.10	0.68	0.09	0.74
